# Overexpression of the apple *SEP1/2*-like gene *MdMADS8* promotes floral determinacy and enhances fruit flesh tissue and ripening

**DOI:** 10.1007/s00425-025-04632-1

**Published:** 2025-02-07

**Authors:** Ling Hoong, Hilary S. Ireland, Sumathi Tomes, Kularajathevan Gunaseelan, Maryam Ruslan, Catherine McKenzie, Ian Hallett, Karine M. David, Robert J. Schaffer

**Affiliations:** 1 The New Zealand Institute for Plant and Food Research Ltd, Auckland Mail Centre, Private Bag 92196, Auckland, 1142 New Zealand; 2 School of Biological Sciences, The University of Auckland, Auckland Mail Centre, Private Bag 92019, Auckland, 1142 New Zealand; 3 The New Zealand Institute for Plant and Food Research Ltd, 412 No 1 Road, TePuke, 3182 New Zealand; 4 The New Zealand Institute for Plant and Food Research Ltd, 55 Old Mill Road, Motueka, Tasman 7198 New Zealand

**Keywords:** Fruit flesh development, MADS-box, *Malus domestica*, *SEPALLATA*

## Abstract

**Main conclusion:**

The over-expression of the apple *SEP1/2* like gene (*MADS8*) supports its role as a key regulator of fleshy fruit patterning and ripening in pome fruit.

**Abstract:**

Fruit flesh patterning in accessory fruits such as apple and strawberry has been associated with the *SEPALLATA* (*SEP*) class of genes. Antisense suppression of multiple *SEP* homologs greatly reduced fruit fleshy tissue growth and ripening in strawberries and apple, suggesting a co-evolution of fleshy fruit growth and its ability to ripen. In the present study, transgenic apple trees were generated that overexpressed the apple *SEP1/2*-like gene *MADS8* (*MADS8ox*). These lines produced precocious flowers in the second year of growth, but subsequent flower numbers were not increased. Overexpression *MdMADS8* in *Arabidopsis* promoted flowering and caused curled leaves and a conversion of sepals to petals. In apple, *MADS8ox* fruit were larger than wild type ‘Royal Gala’ fruit with a higher proportion of flesh tissue compared to core tissue. *MADS8ox* fruit ripened earlier and faster with higher expression of ripening-related genes such as *ACS1* and *ACO1*. Together, these results add further insights into the role that *SEP-*like genes have in controlling flowering and fruit patterning and provide further evidence that *MdMADS8* is one of the key regulators of fruit flesh development and ripening in apples.

**Supplementary Information:**

The online version contains supplementary material available at 10.1007/s00425-025-04632-1.

## Introduction

In angiosperms there is a large diversity of fruiting structures, which have been categorised into either dry fruiting or fleshy fruiting structures. The edible flesh tissue, used to attract seed dispersers, can be derived from different tissues. In most species the flesh is carpel-derived, while in other fruits it is derived from accessory tissue adjacent to the ovary tissue (Karlova et al. 2014). The Rosaceae family, which includes species of economic importance such as almond, strawberry, peaches, cherries, apple and pear, contains a diverse array of fleshy fruit types. Peach and cherry fruits derive from the carpel, while in other species the flesh of the fruit derives from the receptacle (e.g. strawberry) or the hypanthium (e.g. pome fruit such as apples) (Shulaev et al. 2008; Liu et al. 2020). While the fruiting types are closely linked to the floral structure, the molecular determination of how these fleshy fruiting structures are formed is still unclear.

In all core eudicots there is a strong conservation of floral patterning, with essentially all flower types having concentric whorls of sepals, petals, stamens and carpels, controlled by the ABCE MADS-box genes (Ferrario et al. 2004). The MADS-box transcription factors form homo- and hetero-tetramers that determine the different floral structures (Honma and Goto 2001). Key to flower determination are the partially redundant E function *SEPALLATA* (*SEP*) class of genes (Pelaz et al. 2000). The *SEP* genes lay the foundations for flower development, as the removal of all *SEP* gene function results in the formation of whorls of leaves in place of floral organs (Ditta et al. 2004), and leaves can be converted into floral structures only when *SEP* genes are expressed in combination with other ABC genes (Pelaz et al. 2001b). The MADS-box genes have been proposed to work in conjunction with the phytohormone auxin to control the progressive development of the floral meristem (Dornelas et al. 2011). This is supported by the observation that the highly expressed dominant negative *SEP3* gene produces a similar floral phenotype to the auxin-related *ettin* (*ett*) mutant (Kaufmann et al. 2009).

Numerous studies in *Arabidopsis thaliana* and other species have shown that the *SEP* genes have a wider role than just floral patterning. Overexpression of native and heterologous *SEP-like* genes can promote flowering and the production of more flowers (Pelaz et al. 2001a; Uimari et al. 2004; Zhao et al. 2006; Zhang et al. 2017; Adal et al. 2021). This flowering phenotype has been suggested to act by modulating the floral induction and homeotic genes (Castillejo et al. 2005; Kaufmann et al. 2009; Zhang et al. 2017). The *SEP*-like genes also play a key role in the control of fleshy fruit ripening, with the tomato *RIPENING INHIBITOR* (*RIN*) gene encoding a *SEP4* class of MADS transcription factors (Vrebalov et al. 2002). *RIN* has recently been proposed to act with other *SEP*-like genes to promote ripening (Wang et al. 2019). The *SEP1/2* class of genes in strawberry (*FaMADS9*), apple (*MdMADS8*) and banana (*MaMADS1* and *MaMADS2*) have been shown to regulate ripening (Seymour et al. 2011; Ireland et al. 2013; Elitzur et al. 2016), and expression studies have suggested that they play a key role in ethylene-mediated ripening in kiwifruit (McAtee et al. 2015). In many fruit species, RIN homologs have been shown to bind and activate the expression of their own gene and the ethylene biosynthesis gene *ACC SYNTHASE* (*ACS*), making them key components of the regulation of ethylene-associated climacteric fruit ripening (Martel et al. 2011; Fujisawa et al. 2013; Ireland et al. 2013; Lü et al. 2018).

Studies of *SEP*-like genes in the accessory fruit of strawberry and apple, using antisense constructs, have shown that suppression of the *SEP1/2* class of genes resulted in a significant reduction of accessory-derived flesh tissue (Seymour et al. 2011; Ireland et al. 2013). In strawberry, suppression of *FaMADS9* led to a range of phenotypes, the most extreme of which was a great reduction in receptacle tissue. In contrast, a more recent study targeting *FaMADS9* using antisense suppression with a more specific target site, did not result in any reduction in flesh tissue. However, in this later study there were still detectable amounts of *FaMADS9* transcript present, which may explain this result (Vallarino et al. 2020). In apple, the *SEP1/2*-like gene *MdMADS8* was used for antisense suppression (*MADS8as*). In lines with greatly reduced flesh tissue, the closely related *MdMADS9* was also strongly suppressed. There also was a reduction in expression of *MdMADS7*, making it unclear as to which gene, or indeed whether all these genes contributed to flesh patterning. In this current study we overexpressed *MdMADS8* (referred to as *MADS8* hereafter) in apple and *Arabidopsis*, to further understand the role of this gene in apple flesh development and ripening.

## Materials and methods

### Development of the overexpression vector and plant transformation

To prepare the overexpression construct, the full CDS of *MADS8* was amplified from floral cDNA (Ireland et al. 2013) using the primer sequences 5’-ATGGGGAGAGGAAGAGTGGAGCTGAA-3’ and 5’-TCAAAGCATCCATCCAGGGATGAAAC-3’ with high fidelity Platinum *Pfx* polymerase (Thermo Fisher Scientific, Auckland, NZ). The cDNA was cloned into pHEX2 under the control of *CaMV35S* promoter using Gateway Cloning (Thermo Fisher Scientific). Twelve independent lines of transgenic *Malus domestica* ‘Royal Gala’ were generated using *Agrobacterium*-mediated transformation (Yao et al. 1995). Shoots were micrografted onto ‘M9’ rootstocks and were grown under glasshouse containment conditions.

*Arabidopsis* transformation was undertaken using the floral dip methodology (Clough and Bent 1998), germinated on MS 1% agar plates containing 50 µg/ml kanamycin. Kan^R^ plants with green leaves and roots were transferred to rock wool plugs and grown under glasshouse conditions.

### Apple tree and fruit assessments

Four weeks after budbreak, apple tree buds were scored for vegetative, or floral state, and the length of outgrowth was also recorded. Apple fruit were assessed as described in (Johnston et al. 2009). Binomial confidence intervals were calculated for each tree (Suppl. Fig. S1), for the proportion of buds that broke out of the total number of buds. Poisson confidence intervals were calculated for each tree, for flower count and branch length using Minitab statistical software (21st edition, 2023 Pennsylvania, USA. www.minitab.com). 84% confidence intervals were used, as non-overlapping 84% confidence intervals indicates statistically significant differences between the means at α = 0.05 (Payton et al. 2003).

### Microtomography

Flowers at the balloon stage of development were fixed in FAA (4% formaldehyde, 5% acetic acid, 50% ethanol) and washed with 70% ethanol prior to infiltration. Flowers were infiltrated with 1% phosphotungstate for 6 days as described by Staedler et al. (2013) and washed with 70% ethanol (EtOH) prior to mounting. After infiltration, flowers were washed three times with 1 mL 70% EtOH and were mounted individually in the pointed tip-Sect. (5 cm) of a 5-mL Eppendorf pipette tip. Inside the pipette tip, samples were immersed in 70% EtOH. Wax was used to seal both the upper and lower end of the pipette tip. Scanning was performed using a SkyScan-1272 high-resolution micro-CT system, 33 kV acceleration voltage, 150 µA source current, 1000 ms exposure time, 0.4° rotation step, 7.4 µm pixel size, and using flat field correction, geometrical correction and no filter. 3D reconstruction was performed using NRecon software ver. 1.6.9.18.

### Microscopy

Floral buds were collected from plants and immediately fixed (2% paraformaldehyde and 0.1% glutaraldehyde in 0.1 M phosphate buffer, pH 7.2). Samples were prepared for resin embedding with three washes of 0.1 M phosphate buffer, pH 7.2, and one wash of distilled water, then dehydrated with an ethanol series of 30%, 50%, 70%, 95% and twice at 100% for 30 min each. Ethanol was replaced with 50:50 LR white resin:ethanol (LR white resin obtained from London Resin Co, Reading, UK) for 30 min at 50 kPa, followed by two replacements of 100% LR white resin over one day. Samples were then placed in fresh LR white resin in gelatine capsules and polymerised overnight at 60 °C. Resin sections were cut at 1 µm on an Ultracut UCT Ultramicrotome (Leica Microscopy Systems Ltd, Heerbrugg, Switzerland) using a diamond knife (Diatome, Hatfield, PA, USA). Sections were transferred to poly-L-lysine coated slides (LabServ, Thermo Fisher Scientific) and dried at 50 °C. Sections were mounted in Citifluor and imaged with UV (excitation 330–380 nm, dichroic mirror 400 nm, emission 410 + nm) on a Nikon Eclipse Ni-E compound fluorescence microscope with a Nikon DS-Ri2 camera.

### Expression analysis

RNA was extracted from 2–3 g of frozen plant tissues as described by Chang et al. (1993). Prior to cDNA synthesis, each RNA sample was further purified by the removal of genomic DNA using TURBO™ DNAse Kit (Thermo Fisher Scientific) supplemented with RNAseOUT™ ribonuclease inhibitor (Thermo Fisher Scientific) as per the manufacturer’s instructions. cDNA was synthesized from DNase-treated RNA samples using SuperScript® VILO™ cDNA Synthesis Kit as per manufacturer’s instructions. Reagents were incubated at 25 °C for 10 min to allow annealing of Oligo ${(dT)}_{20}$ primers to the poly-A tails of RNA transcripts. Following this, reverse transcription occurred at 42 °C for 60 min and was terminated by heating the reaction mix to 85 °C for 5 min. All cDNA samples were then diluted to 1:10 ratio with ultrapure water (NANOpure® Diamond™) and this diluted cDNA was used as the stock solution for qPCR assays. qPCR reactions using primers (Table 1) were undertaken in a Roche LightCycler® 480 and expression data were analysed using Roche analytical software.

**Table 1:** qPCR primers

Gene	Gene model	Forward primer	Reverse primer	Gene
*MdActin*	MD12G1140800	TGACCGAATGAGCAAGGAAATTACT	TACTCAGCTTTGGCAATCCACATC	*MdActin*
*MdMADS8*	MD17G1065400	AATCATGGGAAGGTGGTGAC	CATCAGCACATGCTCAAAGC	*MdMADS8*
*MdACS1*	MD15G1203500	TCTGTTGGGTGGATATGAGGC	GCATTGCCAAGTCCAGAGT TC	*MdACS1*
*MdACO1*	MD10G1328100	ACCCAGGCAACGACTCATTC	GCAACAGGGGTGGATT CCT	*MdACO1*
*MdPG1*	MD10G1179100	TGAACACTTTGCAGCACGAT	GGCGGTTCAAGTGAAAAATG	*MdPG1*

## Results

### Generation of independent lines overexpressing *MADS8*

**Fig. 1:**
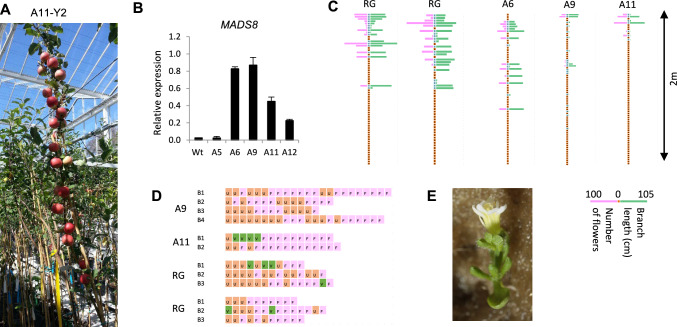
Over expression of *MADS8* promotes precocious flowering and changes tree architecture. **A** *MADS8ox* (line A11) in the second season showing fruit on every node. **B** Expression values of *MADS8* in the leaves of 5 independent lines, relative to *ACTIN.* Values are averages ± SE (*n* = 4). **C** Tree architecture in the third season demonstrating a change in architecture with less shorter branches (green bars) and more blank wood (unbroken buds – orange) in the extreme lines (A9 and A11) number of flowers nodes (pink) are shown. **D** Floral numbers on branches: orange unbroken (U), green vegetative (V), and pink floral (F). **E** Overexpression of *MdMADS8* in *Arabidopsis* promotes flowering and causes curled rosette leaves

To further understand the role of apple *MADS8* in the control of plant development, 12 independent transgenic overexpression lines of ‘Royal Gala’ were generated containing the apple *MADS8* gene controlled by a *CaMV35S* promoter (*MADS8ox*). These were micrografted onto ‘M9’ rootstocks, with five of these lines surviving and growing vegetatively during the first season. In the second season, four of the *MADS8ox* lines appeared more floral than untransformed wild type ‘Royal Gala’ trees, particularly with one (line A11) producing flowers and fruit at every axillary meristem on the primary shoot. This was a particularly striking phenotype, given that micrografted trees rarely flower in the second season (Fig. 1A, Suppl. Fig. S1). Expression analysis of young leaves showed that four lines (A6, A9, A11 and A12) had significantly higher expression of *MADS8* than untransformed (wild type) plants (Fig. 1B). At the end of the second year, all plants were pruned to 2 m height.

Owing to the increased floral phenotype observed in the second year of growth of some of the lines, a detailed phenotypic study was undertaken in the third year. At this point the control plants were also floral, with a similar percentage of floral buds (Fig. 1C, Suppl. Fig. S1). Apples meristems can either stay dormant (do not break bud) or produce an outgrowth (budbreak). The outgrowth can be either vegetative (whorls of leaves) or floral (clusters of flowers plus leaves) (Fig. 1C). The wild-type control plants showed a balance of floral and vegetative growth along the length of the primary shoot, while in two extreme *MADS8ox* lines (A9 and A11) most of the axillary buds (that had been floral in the previous year) did not break. Those that did were at the apical region, and were shorter than the untransformed apples (Suppl. Fig. S1), but in this and subsequent years the branches were not significantly more floral than the wild type untransformed apples. (Fig. 1D).

To further investigate the floral phenotype, the *MdMADS8* over-expression construct was transformed into *Arabidopsis*. Plants with high expression of *MdMADS8* (Suppl. Fig. S2) had curled rosette leaves and early flowering with reduced number of rosette leaves compared to untransformed control (Fig. 1E, Suppl. Fig. S2) further supporting the role of *MdMADS8* in promoting flowering. Additionally, the floral organ identity in *Arabidopsis* was also altered with intermediate phenotypes showing an extra petal, and in extreme phenotypes there was a conversion of the sepals to petals. This increase in petal determinacy was consistent with the petal to sepal conversion observed in the *MADS8* antisense plants (Ireland et al 2013).

### Effect of *MADS8* on floral structure

**Fig. 2:**
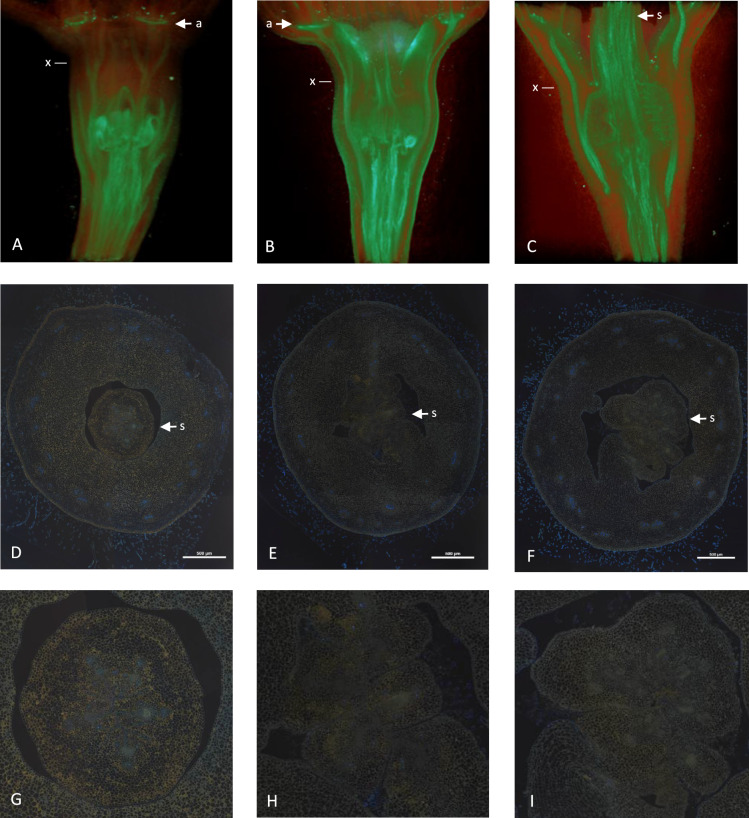
3D microtomography images and sections showing vasculature in open apple flowers of wild type (**A**, **D**, **G**), *MADS8ox* (line A11) (**B**, **E**, **H**), and *MADS8as* (**C**, **F**, **I**). **A–C** Microtomography, with the abscission zones (arrow a). **D–I** UV fluorescence of cross sections at the point where the stylar tissue fuses with the hypanthium (line marked with x). Lignified xylem tissue fluoresces blue. **G–I** Magnified sections of the stylar tissue (arrow s). Magnification bar = 500 µm

As reported previously, there appeared to be a change in the vascular arrangement of the flowers in the *MADS8as* lines, as well as the loss of flesh tissue (Ireland et al. 2013). To assess changes in vascular structures in flowers from the *MADS8as*, wild type and *MADS8ox* lines, floral buds were infiltrated with phosphotungstate and visualised with 3D X-ray microtomography (Staedler et al. 2013). Phosphotungstate was chosen as it has high affinity to vasculature, cytoplasmic-rich tissue and pollen (Staedler et al. 2013). The microtomography images showed an increased signal within the stylar tissue(s) in the *MADS8as* lines. It also highlighted the loss of the petal abscission zone (a) in these lines compared with the wild type and *MADS8ox* lines (Fig. 2A–C, arrows). To further investigate these floral differences, standard cross sections of open flowers were taken at the point where the stylar ovary tissue fuses to the hypanthium and UV florescence was used to observe xylem tissue in the vasculature (Fig. 2D–I). The stylar tissue in wild type apple appeared as a round fused structure at the fusion boundary, with five vascular bundles. Both the *MADS8ox* and *MADS8as* lines showed a style with poor fusion and disrupted patterning. It was unclear whether the increased uptake of phosphotungstate by the *MADS8as* lines resulted from an increased vasculature tissue or an other tissue, but there was a clear difference in structure.

### The effect of *MADS8* on cortex flesh tissue

**Fig. 3:**
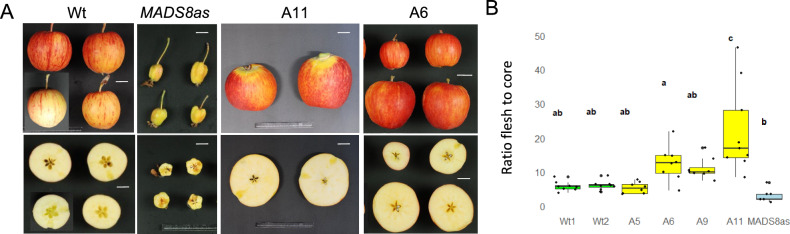
Increased flesh tissue in the *MADS8* overexpression lines. **A** Royal gala wild type (Wt), *MADS8* antisense (*MADS8as*), and representative *MADS8* overexpression lines A11 and A6. **B** Box plots of ratio of fruit flesh to core tissue of Wt (green), 4 *MADS8* overexpression lines (yellow) and *MADS8as* (blue). Letters represent Tukey’s Honestly Significant Difference test *P* < 0.05 (*n* = 8), following one way ANOVA

To assess the effect of *MADS8ox* on flesh development, mature apple fruit from wild type and *MADS8ox* lines were cut transversely (Fig. 3A) and the distance from the centre of the apple to the edge of the core (core tissue), and the distance from the edge of the core to the skin was measured (flesh tissue). The vascular bundles located at the end of the locules were used to demarcate the edge of the core tissue. Owing to large variation of fruit size, which is the results of both variation of flesh and core tissue, the ratio of flesh to core tissue was used. It was found that wild type apples typically had a ratio of 5:1 flesh to core. Over expression lines A6, A9 and A11 had a higher ratio of flesh to core, with A11 demonstrating a significant difference with an average ratio of 23:1 (Fig. 3B).

### The effect of *MADS8* on fruit ripening

**Fig. 4:**
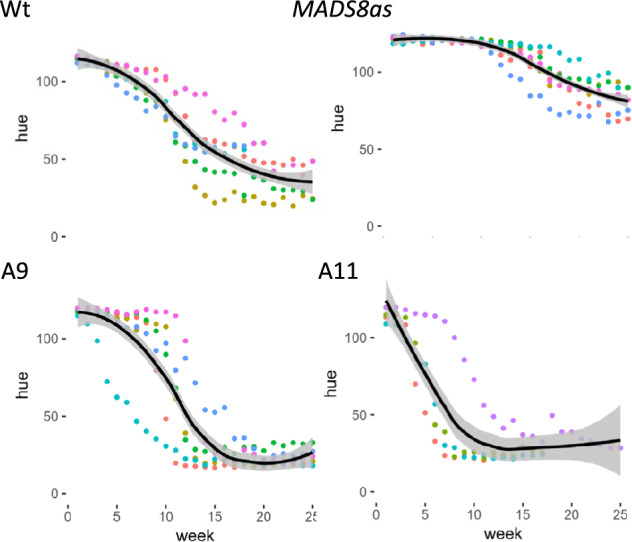
Background skin colour progression in individual fruits (each a different colour) from wild-type (Wt), transgenic *MADS8ox* (A9 and A11), and *MADS8as* lines between 80 days after full bloom (DAFB) (week 1) and 172 DAFB (week 25) or until fruit-fall. Loess smoothing curves are shown in black, with grey shading showing the 95% confidence intervals. Hue angles were recorded for upto six individual fruits from each line and found to progress from green, to yellow. Data from all lines are shown in additional data

The *MADS8as* lines did not undergo climacteric-associated fruit ripening (Ireland et al. 2013). To assess whether overexpression of *MADS8* had any effect on the speed of ripening, we assessed different ripening attributes in the overexpression and antisense lines. Background skin colour was initially used to assess the progression of ripening since this non-destructive trait can be easily followed during development and ripening. In apple, the background green colour changes to yellow (degreening) as the fruit ripens, independently of the red blush, which is formed earlier in fruit development. In wild type apples the background colour started degreening at about 118 days after full bloom. In two of the lines (A6 and A9) the colour change started at a similar time, but occurred faster than in the wild type (Fig. 4, Supl. Fig. S3). In the more extreme line A11 the background colour change occurred earlier than in the wild type, as well as being faster. Consistent with previous results, the *MADS8as* lines had very little colour change during this period (Ireland et al. 2013).

**Fig. 5:**
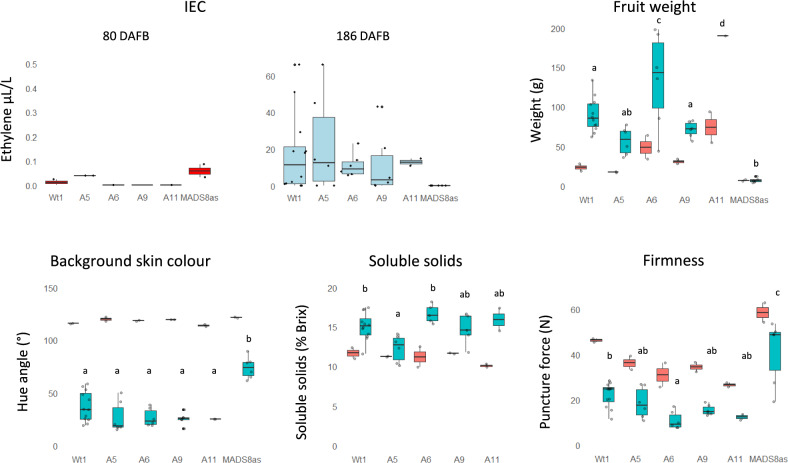
Ripening-related physiological assessment of wild-type, and transgenic *MADS8ox* and *MADS8as* lines at 80 DAFB (red) and at 186 DAFB or fruit-fall (blue): IEC (internal ethylene concentration), fruit weight, soluble solids concentration, background skin colour and firmness. Due to limitations of apple numbers for 80 DAFB only 2 apples were sampled. For 186 DAFB 2–6 apples were assessed. Letters represent Tukey’s Honestly Significant Difference test *P* < 0.05, following one way ANOVA

Other ripening characters that required destructive measurements was limited by small numbers of fruit. Two time points were chosen, an early harvest (80 days after full bloom (DAFB)) and a late harvest (186 DAFB). At the early harvest, the internal ethylene concentration was very low (close to the detection limit) in wild type, A5 and *MADS8as* apples, and undetectable in the A6, A9 and A11 lines (Fig. 5A). As the fruit ripened there was an increase in internal ethylene in all lines except the *MADS8as.* The fruit of A11 and A6 lines showed a significantly larger apples, though the total crop for these trees were lower. At the late harvest the *MADSox* lines background skin colour was statistically similar to the wild type, and the *MADS8as* did not degreen as much. At 186 DAFB line A6, A9 and A11 were slightly softer, with A6 having significant softer apples than the wild type (Fig. 5). The soluble solids contents were similar in all lines, except the *MADS8as* lines, which could not be tested because of lack of free juice. Overall, these data suggests that the end-point ripening of the overexpression apples were not considerably different from wild type Royal Gala.

### Ripening associated genes

Although under a constitutive *CaMV35S* promoter, it was found that the *MADS8* transcript in maturing and ripening fruit appeared to be post transcriptionally regulated. In the lines having higher expression in the leaves the mRNA content was similar to wild type untransformed apples in maturing and ripening fruit (Suppl. Fig. S4). At 80 DAFB when the fruit were undergoing maturation, most lines (except A11), had a similar *MADS8* expression. By 186 DAFB, the fruit had ripened on the tree, and expression of *MADS8* gene in the wild type and in all transgenic lines being very low (Suppl. Fig. S4). At this late time point, the *ACS1* and *ACO1* gene expressions, which have previously been shown to be direct targets of MADS8 (Ireland et al. 2013), were increased only in two lines (A6 and A11) (Suppl. Fig. S4).

## Discussion

Consistent with the proposed model of coevolution of flesh development and ripening in apple (Ireland et al. 2013), this study further supports a central role of apple *MADS8* in flesh patterning and fruit ripening. It was found that increasing expression of *MADS8* resulted in relatively more flesh tissue and faster ripening. Combined with data from previous studies of *MADS8as* plants that showed less flesh and slower ripening compared with the wild type ‘Royal Gala’ (Ireland et al. 2013), this suggests that *MADS8* acts in a dose-dependent manner in controlling the amount of apple flesh and the speed of ripening in different apple varieties. However, as there is a reduction in the overall expression of MADS8 as the apple develops, it suggests that this gene is tightly controlled at the transcript level, especially at fruit maturation and ripening. We speculate that small increases at this time can affect speed and maturation of ripening as observed, even though transcriptionally there were little differences in expression.

It has previously been shown that the *SEPALLATA* genes are highly functionally redundant, with single mutations often not showing strong phenotypes (Pelaz et al. 2000; Ditta et al. 2004). Combinations of the *sep* mutations have been used to reveal specific functions for the different classes of *SEP* genes, but it is only when all the genes have been suppressed, or when using dominant negative mutations, that the complete functions of the *SEP* clade of genes are revealed (Pelaz et al. 2000; Vrebalov et al. 2002; Ditta et al. 2004). In apple, the *MADS8as* construct was shown to strongly down-regulate the close homologue *MADS9* as well as reducing the expression of a few other *SEP-*like genes (Ireland et al. 2013). This meant it was unclear whether *MADS8* or *MADS9* individually controlled apple fruit flesh development and ripening, and whether these genes acted redundantly with other *SEP-*like genes. Despite the general appearance of redundancy in *A. thaliana*, in other species the *SEP* genes, in terms of their regulation of floral identity, do appear to have evolved more specialised roles in fruit development. Recently it was shown that *SEP1/2*-like genes from fleshy accessory-fruiting Rosaceae species are under positive selection pressure compared with those from true-fruiting Rosaceae species such as dry fruit-bearing *Gillenia trifoliata* and drupaceous *Prunus* species, supporting their role in accessory flesh development (Ireland et al. 2021b). Dry to fleshy fruit with ripening appear to have coevolved in apple, with MADS9 appearing to be the more dominant ripening regulator (Ireland et al. 2013); as such, both *MADS8* and *MADS9* were under positive selection (Ireland et al. 2021b). Here, specific overexpression of *MADS8* resulted in enhanced fruit flesh growth, but this does not rule out the possibility that *MADS9* may also act redundantly with *MADS8* to control flesh development.

Further specialisation of apple *MADS8* includes setting up abscission zones in the petals and the control of carpel fusion at the styles. Of these two more subtle phenotypes, the lack of petal abscission was reported by (Ireland et al. 2013), with the green petals being maintained throughout apple fruit development. The lack of an abscission zone in the *MADS8as* line is clearly seen in the X-ray microtomography images (Fig. 2C). Abscission zone development is associated with a loss of auxin flux, further strengthening the link between *SEP* genes and auxin signalling (Dornelas et al. 2011). The lack of stylar fusion occurring in both the overexpression lines and the antisense lines is of interest and warrants further investigation.

Overexpression of *SEP* genes in other species, and in heterologous systems, often results in a decrease in time to flower and increase in floral numbers (Pelaz et al. 2001a; Zhao et al. 2006; Zhang et al. 2017; Adal et al. 2021). Here we have shown that this function is conserved in the woody species apple, with apple *SEP1/2-*like genes increasing floral transition of meristems early in development. The floral promotion of this class of genes in apple was further supported by precocious flowering in *Arabidopsis* with this construct. The highly floral phenotype of the MADS8ox apples appears to stop further meristem outgrowth, resulting in long stretches of stem having ‘blind wood’ where no budbreak occurs. As no significant increase in floral buds were observed in subsequent years it would appear that *MADS8* cannot further enhance flowering beyond the first year’s growth. This is a subtle effect on flowering in apples when compared to apple studies that use other floral promoters such as those using the AP1 clade gene *BpMADS4* gene (Flachowsky et al. 2007). In these plants the annual flowering is completely bypassed, with every flush of growth containing flowers. The *MADS8ox* floral phenotype maintained the annual flowering cycle, with no flowers observed in the first year of growth, a strong promotion of flowering in the second, and little promotion of flowering beyond that and no observed flowering out of the spring flowering cycle.

Typically, consumers throw away the core of the apples, thus a way of removing or reducing the core in apples could increase convenience (and reduce waste) (Ireland et al. 2021a). This research suggests an approach to reduce the core component of apples might be to upregulate the *MADS8* gene. This would provide a higher proportion of flesh tissue with less food waste. However, a flower-specific promoter would be preferable to the constitutive *CaMV35S* promoter used in this study, as increased floriferousness in one growing season resulted in poor return bloom and blind (non-sprouting) sections of wood in the next growing season. A flower-specific promoter would also avoid higher *MADS8* expression later in fruit development, which would lead to faster ripening.

## Supplementary Information

Below is the link to the electronic supplementary material.

Supplementary file1 (PPTX 733 KB)

Supplementary file2 (PPTX 492 KB)

Supplementary file3 (PPTX 536 KB)

Supplementary file4 (PPTX 51 KB)

## Data Availability

All data generated or analysed during this study are included in this published article.

## Abbreviations

ACS: *ACC SYNTHASE*
DAFB: Days after full bloom
*SEP*: *SEPALLATA*

## References

[CR1] Adal AM, Binson E, Remedios L, Mahmoud SS (2021) Expression of lavender *AGAMOUS*-like and *SEPALLATA3*-like genes promote early flowering and alter leaf morphology in *Arabidopsis thaliana*. Planta 254(3):1–12 34410495 10.1007/s00425-021-03703-3

[CR2] Castillejo C, Romera-Branchat M, Pelaz S (2005) A new role of the Arabidopsis *SEPALLATA3* gene revealed by its constitutive expression. Plant J 43(4):586–596 16098111 10.1111/j.1365-313X.2005.02476.x

[CR3] Chang S, Puryear J, Cairney J (1993) A simple and efficient method for isolating RNA from pine trees. Plant Mol Biol Rep 11(2):113–116

[CR4] Clough SJ, Bent AF (1998) Floral dip: a simplified method for *Agrobacterium*-mediated transformation of *Arabidopsis thaliana*. Plant J 16(6):735–743 10069079 10.1046/j.1365-313x.1998.00343.x

[CR5] Ditta G, Pinyopich A, Robles P, Pelaz S, Yanofsky MF (2004) The *SEP4* gene of *Arabidopsis thaliana* functions in floral organ and meristem identity. Curr Biol 14(21):1935–1940 15530395 10.1016/j.cub.2004.10.028

[CR6] Dornelas MC, Patreze CM, Angenent GC, Immink RG (2011) MADS: the missing link between identity and growth? Trends Plant Sci 16(2):89–97 21144794 10.1016/j.tplants.2010.11.003

[CR7] Elitzur T, Yakir E, Quansah L, Zhangjun F, Vrebalov J, Khayat E, Giovannoni JJ, Friedman H (2016) Banana *MaMADS* transcription factors are necessary for fruit ripening and molecular tools to promote shelf-life and food security. Plant Physiol 171(1):380–391. 10.1104/pp.15.01866 26956665 10.1104/pp.15.01866 PMC4854685

[CR8] Ferrario S, Immink RG, Angenent GC (2004) Conservation and diversity in flower land. Curr Opin Plant Biol 7(1):84–91 14732446 10.1016/j.pbi.2003.11.003

[CR9] Flachowsky H, Peil A, Sopanen T, Elo A, Hanke V (2007) Overexpression of BpMADS4 from silver birch (Betula pendula Roth.) induces early-flowering in apple (Malus× domestica Borkh.). Plant Breed 126(2):137–145

[CR10] Fujisawa M, Nakano T, Shima Y, Ito Y (2013) A large-scale identification of direct targets of the tomato MADS box transcription factor RIPENING INHIBITOR reveals the regulation of fruit ripening. Plant Cell 25(2):371–386 23386264 10.1105/tpc.112.108118 PMC3608766

[CR11] Honma T, Goto K (2001) Complexes of MADS-box proteins are sufficient to convert leaves into floral organs. Nature 409(6819):525–529 11206550 10.1038/35054083

[CR12] Ireland HS, Yao JL, Tomes S, Sutherland PW, Nieuwenhuizen N, Gunaseelan K, Winz RA, David KM, Schaffer RJ (2013) Apple *SEPALLATA1/2*-like genes control fruit flesh development and ripening. Plant J 73(6):1044–1056 23236986 10.1111/tpj.12094

[CR13] Ireland HS, Tomes S, Hallett IC, Karunairetnam S, David KM, Yao J-L, Schaffer RJ (2021a) Coreless apples generated by the suppression of carpel genes and hormone-induced fruit set. Fruit Res 1(1):1–9

[CR14] Ireland HS, Wu C, Deng CH, Hilario E, Saei A, Erasmuson S, Crowhurst RN, David KM, Schaffer RJ, Chagné D (2021b) The *Gillenia trifoliata* genome reveals dynamics correlated with growth and reproduction in Rosaceae. Hort Res 8:233 10.1038/s41438-021-00662-4 PMC8558331 34719690

[CR15] Johnston JW, Gunaseelan K, Pidakala P, Wang M, Schaffer RJ (2009) Co-ordination of early and late ripening events in apples is regulated through differential sensitivities to ethylene. J Exp Bot 60(9):2689–2699 19429839 10.1093/jxb/erp122 PMC2692014

[CR16] Karlova R, Chapman N, David K, Angenent GC, Seymour GB, de Maagd RA (2014) Transcriptional control of fleshy fruit development and ripening. J Exp Bot 65(16):4527–4541 25080453 10.1093/jxb/eru316

[CR17] Kaufmann K, Muino JM, Jauregui R, Airoldi CA, Smaczniak C, Krajewski P, Angenent GC (2009) Target genes of the MADS transcription factor *SEPALLATA3*: integration of developmental and hormonal pathways in the *Arabidopsis* flower. PLoS Biol 7(4):e1000090 19385720 10.1371/journal.pbio.1000090 PMC2671559

[CR18] Liu Z, Ma H, Jung S, Main D, Guo L (2020) Developmental mechanisms of fleshy fruit diversity in Rosaceae. Annu Rev Plant Biol 71(1):547–573 32442388 10.1146/annurev-arplant-111119-021700

[CR19] Lü P, Yu S, Zhu N, Chen Y-R, Zhou B, Pan Y, Tzeng D, Fabi JP, Argyris J, Garcia-Mas J (2018) Genome encode analyses reveal the basis of convergent evolution of fleshy fruit ripening. Nat Plants 4(10):784–791 30250279 10.1038/s41477-018-0249-z

[CR20] Martel C, Vrebalov J, Tafelmeyer P, Giovannoni JJ (2011) The tomato MADS-box transcription factor RIPENING INHIBITOR interacts with promoters involved in numerous ripening processes in a COLORLESS NONRIPENING-dependent manner. Plant Physiol 157(3):1568–1579 21941001 10.1104/pp.111.181107 PMC3252172

[CR21] McAtee PA, Richardson AC, Nieuwenhuizen NJ, Gunaseelan K, Hoong L, Chen X, Atkinson RG, Burdon JN, David KM, Schaffer RJ (2015) The hybrid non-ethylene and ethylene ripening response in kiwifruit (*Actinidia chinensis*) is associated with differential regulation of MADS-box transcription factors. BMC Plant Biol 15(1):1–16 26714876 10.1186/s12870-015-0697-9 PMC4696264

[CR22] Payton ME, Greenstone MH, Schenker N (2003) Overlapping confidence intervals or standard error intervals: what do they mean in terms of statistical significance? J Insect Sci 3(1):34 15841249 10.1093/jis/3.1.34 PMC524673

[CR23] Pelaz S, Ditta GS, Baumann E, Wisman E, Yanofsky MF (2000) B and C floral organ identity functions require *SEPALLATA* MADS-box genes. Nature 405(6783):200–203 10821278 10.1038/35012103

[CR24] Pelaz S, Gustafson-Brown C, Kohalmi SE, Crosby WL, Yanofsky MF (2001a) *APETALA1* and *SEPALLATA3* interact to promote flower development. Plant J 26(4):385–394 11439126 10.1046/j.1365-313x.2001.2641042.x

[CR25] Pelaz S, Tapia-López R, Alvarez-Buylla ER, Yanofsky MF (2001b) Conversion of leaves into petals in *Arabidopsis*. Curr Biol 11(3):182–184 11231153 10.1016/s0960-9822(01)00024-0

[CR26] Seymour GB, Ryder CD, Cevik V, Hammond JP, Popovich A, King GJ, Vrebalov J, Giovannoni JJ, Manning K (2011) A SEPALLATA gene is involved in the development and ripening of strawberry (Fragaria× ananassa Duch.) fruit, a non-climacteric tissue. J Exp Bot 62(3):1179–1188 21115665 10.1093/jxb/erq360 PMC3022409

[CR27] Shulaev V, Korban SS, Sosinski B, Abbott AG, Aldwinckle HS, Folta KM, Iezzoni A, Main D, Arús P, Dandekar AM, Lewers K, Brown SK, Davis TM, Gardiner SE, Potter D, Veilleux RE (2008) Multiple models for Rosaceae genomics. Plant Physiol 147(3):985–1003 18487361 10.1104/pp.107.115618 PMC2442536

[CR28] Staedler YM, Masson D, Schönenberger J (2013) Plant tissues in 3D via X-ray tomography: simple contrasting methods allow high resolution imaging. PLoS ONE 8(9):e75295 24086499 10.1371/journal.pone.0075295 PMC3785515

[CR29] Uimari A, Kotilainen M, Elomaa P, Yu D, Albert VA, Teeri TH (2004) Integration of reproductive meristem fates by a *SEPALLATA*-like MADS-box gene. Proc Nat Acad Sci USA 101(44):15817–15822 15505223 10.1073/pnas.0406844101 PMC524820

[CR30] Vallarino JG, Merchante C, Sánchez-Sevilla JF, de Luis Balaguer MA, Pott DM, Ariza MT, Casanal A, Posé D, Vioque A, Amaya I (2020) Characterizing the involvement of *FaMADS9* in the regulation of strawberry fruit receptacle development. Plant Biotech J 18(4):929–943 10.1111/pbi.13257 PMC7061862 31533196

[CR31] Vrebalov J, Ruezinsky D, Padmanabhan V, White R, Medrano D, Drake R, Schuch W, Giovannoni J (2002) A MADS-box gene necessary for fruit ripening at the tomato *Ripening-Inhibitor* (*Rin*) locus. Science 296(5566):343–346 11951045 10.1126/science.1068181

[CR32] Wang R, da Rocha Tavano EC, Lammers M, Martinelli AP, Angenent GC, de Maagd RA (2019) Re-evaluation of transcription factor function in tomato fruit development and ripening with CRISPR/Cas9-mutagenesis. Sci Rep 9(1):1696 30737425 10.1038/s41598-018-38170-6 PMC6368595

[CR33] Yao J-L, Cohen D, Atkinson R, Richardson K, Morris B (1995) Regeneration of transgenic plants from the commercial apple cultivar Royal Gala. Plant Cell Rep 14(7):407–412 24185446 10.1007/BF00234044

[CR34] Zhang S, Lu S, Yi S, Han H, Liu L, Zhang J, Bao M, Liu G (2017) Functional conservation and divergence of five *SEPALLATA*-like genes from a basal eudicot tree. Platanus Acerifolia Planta 245(2):439–457 27833998 10.1007/s00425-016-2617-0

[CR35] Zhao XY, Cheng ZJ, Zhang XS (2006) Overexpression of *TaMADS1*, a *SEPALLATA*-like gene in wheat, causes early flowering and the abnormal development of floral organs in *Arabidopsis*. Planta 223(4):698–707 16177912 10.1007/s00425-005-0123-x

