# Supplementary material for: Overexpression of the apple SEP1/2-like gene MdMADS8 promotes floral determinacy and enhances fruit flesh tissue and ripening
Source: Planta. 2025 Feb 7;261(3):53. doi: 10.1007/s00425-025-04632-1 (PMC11805781; doi:10.1007/s00425-025-04632-1)
Supplement: Supplementary file 1 — Supplementary file1 (PPTX 733 KB) [file 425_2025_4632_MOESM1_ESM.pptx]

## Slide 1
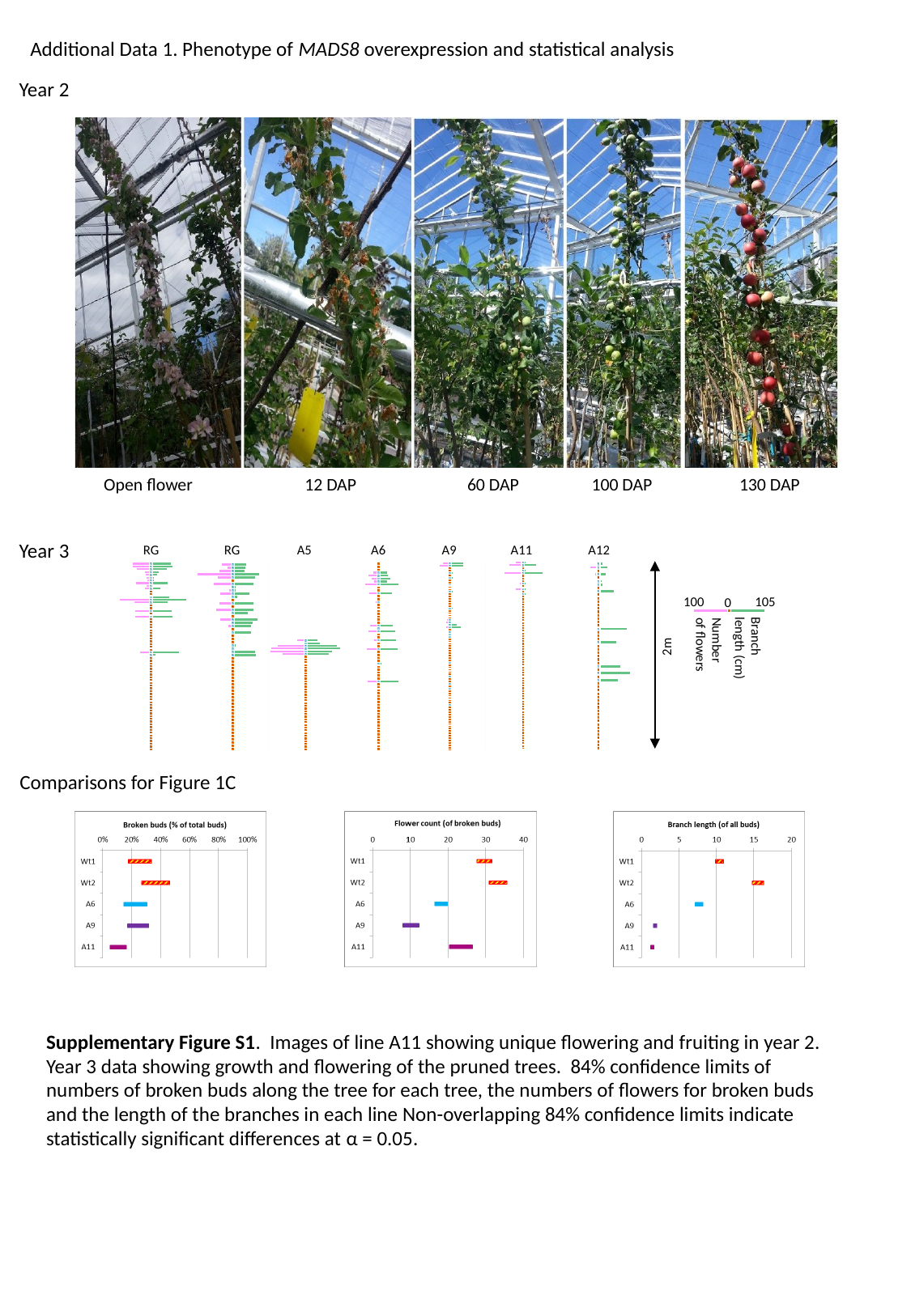

Additional Data 1. Phenotype of MADS8 overexpression and statistical analysis
Year 2
Open flower
12 DAP
60 DAP
100 DAP
130 DAP
Year 3
RG
RG
A5
A6
A9
A11
A12
100
105
0
Number of flowers
Branch length (cm)
2m
Comparisons for Figure 1C
Supplementary Figure S1. Images of line A11 showing unique flowering and fruiting in year 2. Year 3 data showing growth and flowering of the pruned trees. 84% confidence limits of numbers of broken buds along the tree for each tree, the numbers of flowers for broken buds and the length of the branches in each line Non-overlapping 84% confidence limits indicate statistically significant differences at α = 0.05.
