# Supplementary material for: Overexpression of the apple SEP1/2-like gene MdMADS8 promotes floral determinacy and enhances fruit flesh tissue and ripening
Source: Planta. 2025 Feb 7;261(3):53. doi: 10.1007/s00425-025-04632-1 (PMC11805781; doi:10.1007/s00425-025-04632-1)
Supplement: Supplementary file 2 — Supplementary file2 (PPTX 492 KB) [file 425_2025_4632_MOESM2_ESM.pptx]

## Slide 1
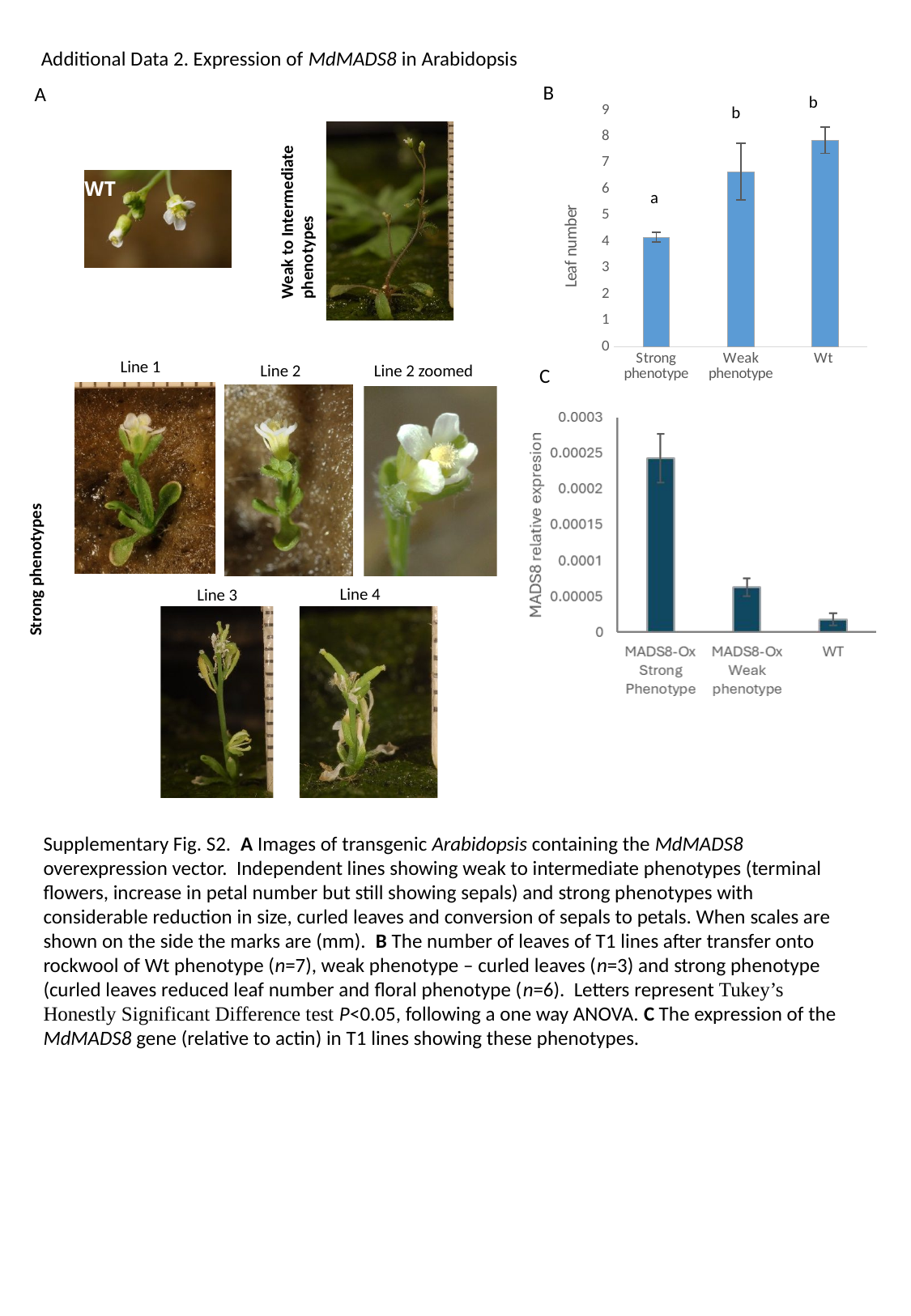

Additional Data 2. Expression of MdMADS8 in Arabidopsis
B
A
b
b
a
### Chart
| Category | |
|---|---|
| Strong phenotype | 4.166666666666667 |
| Weak phenotype | 6.666666666666667 |
| Wt | 7.857142857142857 |
WT
Weak to Intermediate phenotypes
Line 1
Line 2 zoomed
Line 2
C
Strong phenotypes
Line 4
Line 3
Supplementary Fig. S2. A Images of transgenic Arabidopsis containing the MdMADS8 overexpression vector. Independent lines showing weak to intermediate phenotypes (terminal flowers, increase in petal number but still showing sepals) and strong phenotypes with considerable reduction in size, curled leaves and conversion of sepals to petals. When scales are shown on the side the marks are (mm). B The number of leaves of T1 lines after transfer onto rockwool of Wt phenotype (n=7), weak phenotype – curled leaves (n=3) and strong phenotype (curled leaves reduced leaf number and floral phenotype (n=6). Letters represent Tukey’s Honestly Significant Difference test P<0.05, following a one way ANOVA. C The expression of the MdMADS8 gene (relative to actin) in T1 lines showing these phenotypes.
