# Supplementary material for: Overexpression of the apple SEP1/2-like gene MdMADS8 promotes floral determinacy and enhances fruit flesh tissue and ripening
Source: Planta. 2025 Feb 7;261(3):53. doi: 10.1007/s00425-025-04632-1 (PMC11805781; doi:10.1007/s00425-025-04632-1)
Supplement: Supplementary file 3 — Supplementary file3 (PPTX 536 KB) [file 425_2025_4632_MOESM3_ESM.pptx]

## Slide 1
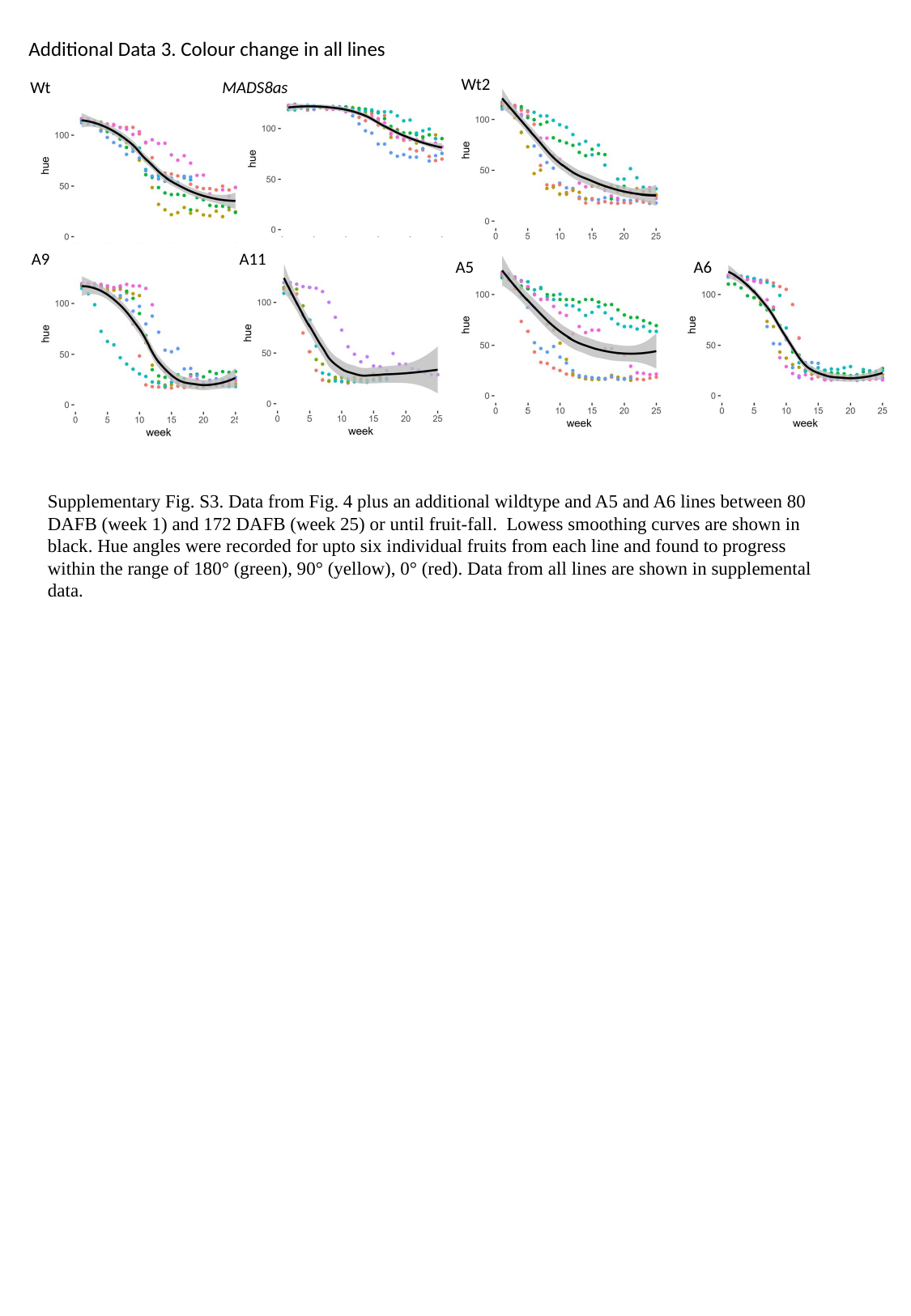

Additional Data 3. Colour change in all lines
Wt2
Wt
MADS8as
A9
A11
A5
A6
Supplementary Fig. S3. Data from Fig. 4 plus an additional wildtype and A5 and A6 lines between 80 DAFB (week 1) and 172 DAFB (week 25) or until fruit-fall. Lowess smoothing curves are shown in black. Hue angles were recorded for upto six individual fruits from each line and found to progress within the range of 180° (green), 90° (yellow), 0° (red). Data from all lines are shown in supplemental data.
