# Supplementary material for: Overexpression of the apple SEP1/2-like gene MdMADS8 promotes floral determinacy and enhances fruit flesh tissue and ripening
Source: Planta. 2025 Feb 7;261(3):53. doi: 10.1007/s00425-025-04632-1 (PMC11805781; doi:10.1007/s00425-025-04632-1)
Supplement: Supplementary file 4 — Supplementary file4 (PPTX 51 KB) [file 425_2025_4632_MOESM4_ESM.pptx]

## Slide 1
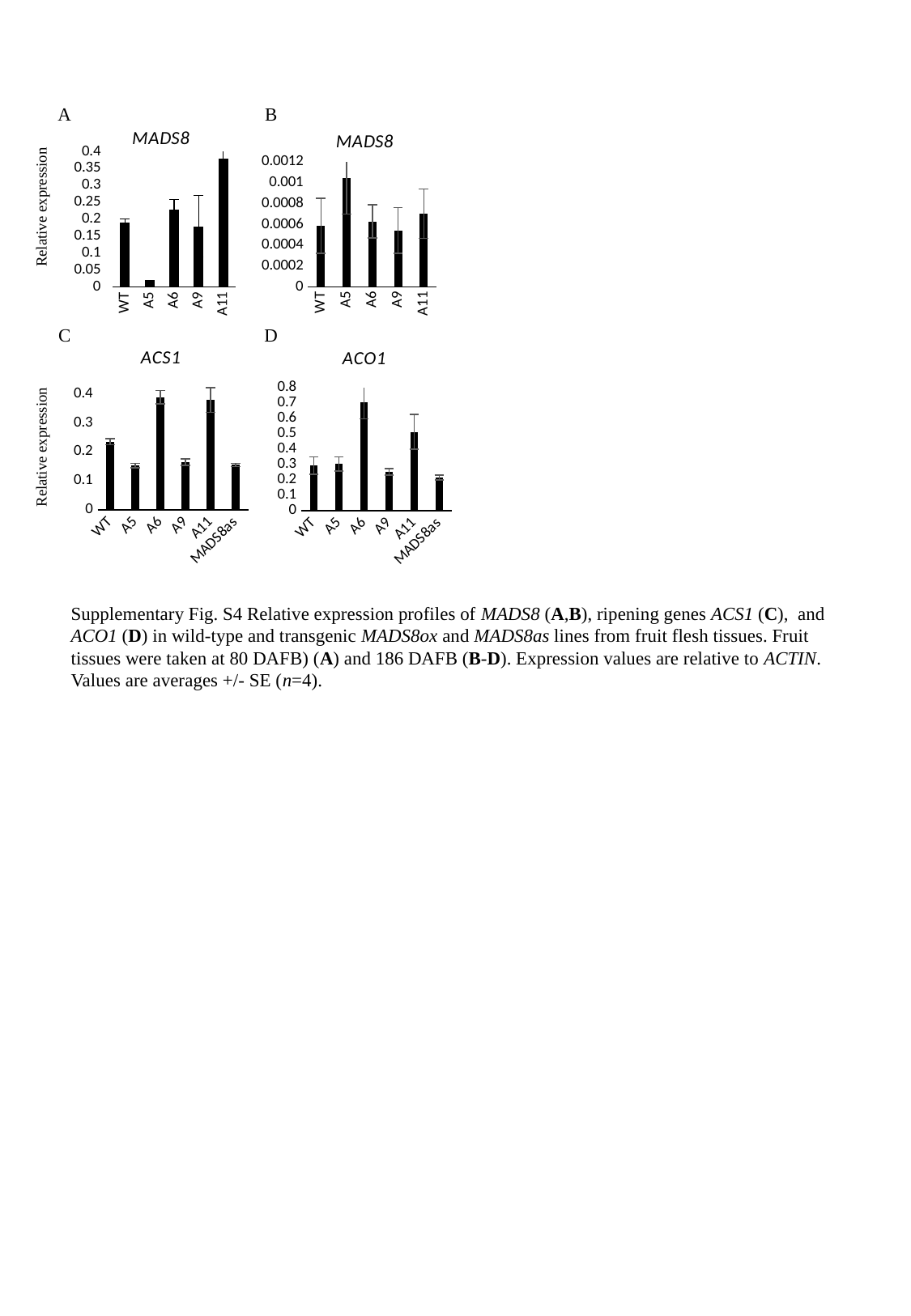

A
B
### Chart: MADS8
| Category | |
|---|---|
| WT | 0.1898 |
| A5 | 0.0171 |
| A6 | 0.2282 |
| A9 | 0.1796 |
| A11 | 0.3788 |
### Chart: MADS8
| Category | |
|---|---|
| WT | 0.00058805 |
| A5 | 0.0010443333333333333 |
| A6 | 0.0006295000000000001 |
| A9 | 0.0005417666666666666 |
| A11 | 0.0007025 |Relative expression
C
D
### Chart: ACS1
| Category | |
|---|---|
| WT | 0.23523333333333332 |
| A5 | 0.15181666666666668 |
| A6 | 0.3880666666666666 |
| A9 | 0.1644 |
| A11 | 0.3792 |
| MADS8as | 0.15446666666666667 |
### Chart: ACO1
| Category | |
|---|---|
| WT | 0.2936166666666667 |
| A5 | 0.3051833333333333 |
| A6 | 0.7043500000000001 |
| A9 | 0.2527833333333333 |
| A11 | 0.5136000000000001 |
| MADS8as | 0.21536666666666662 |Relative expression
Supplementary Fig. S4 Relative expression profiles of MADS8 (A,B), ripening genes ACS1 (C), and ACO1 (D) in wild-type and transgenic MADS8ox and MADS8as lines from fruit flesh tissues. Fruit tissues were taken at 80 DAFB) (A) and 186 DAFB (B-D). Expression values are relative to ACTIN. Values are averages +/- SE (n=4).
